# A Novel Fasciocutaneous Flap Design for Reconstructions in Scarred Tissue: A Case Report

**DOI:** 10.7759/cureus.16402

**Published:** 2021-07-15

**Authors:** Nishank Manohar, Madhubari Vathulya, Sameer Mahakalkar

**Affiliations:** 1 Burns and Plastic Surgery, All India Institute of Medical Sciences, Rishikesh, Rishikesh, IND

**Keywords:** lower extremity, knee joint, pressure injury, scarred tissue, soft-tissues reconstruction, reconstructive flap surgery, forearm fascia defect

## Abstract

This article aims to introduce a technically easy and reliable flap design for the coverage of soft tissue defects in areas where traditional flap options are limited by trauma or scarring. We applied the boomerang design in cases with defects around the knee and also extrapolated it to other regions like the wrist and sacrum. Patients with soft tissue defects in regions with scarred tissues or limited flap reconstructive options were recruited. The procedures resulted in uneventful recovery and excellent cosmetic outcomes for the patients. The authors of this article recommend the usage of this uncomplicated flap design in areas with otherwise limited flap options due to restricted vascularity or surrounding scar tissue.

## Introduction

Fasciocutaneous flaps have traditionally been used as workhorse flaps for soft tissue defect coverage. However, reconstructive surgeons often face difficulty in raising these flaps in the setting of extensive scarring owing to trauma, burns, etc., or due to previous incision lines. We present a modified flap design for reconstruction using local perforator-based fasciocutaneous flaps rotated from around the defect in a boomerang fashion for the reconstruction of such defects. The term boomerang here refers to the shape of the flap. This provides stable coverage of the defect by a technically simple procedure with minimal morbidity among the patients. We share our experience using this novel flap design that led to favorable outcomes.

## Case presentation

Case 1

**Figure 1:**
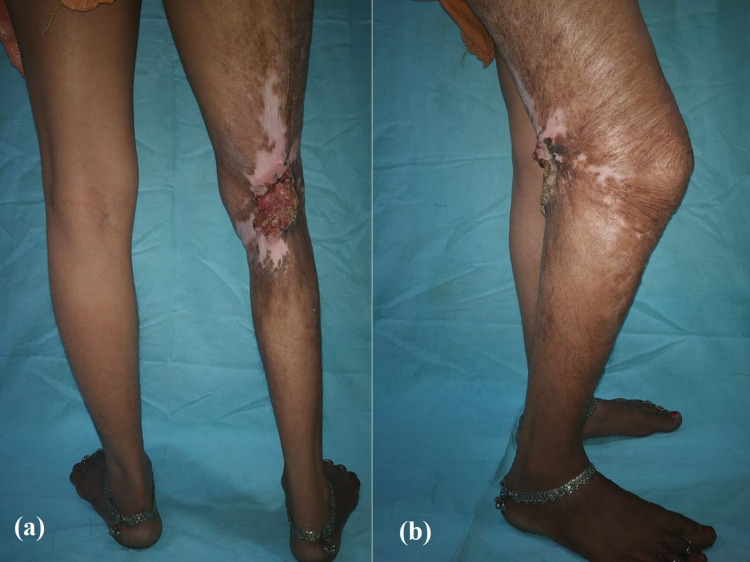
Preoperative photographs of case 1 Clinical photographs showing extensive post-burn scarring in the right popliteal fossa with a restricted range of motion (a) posterior aspect; (b) lateral aspect

**Figure 2:**
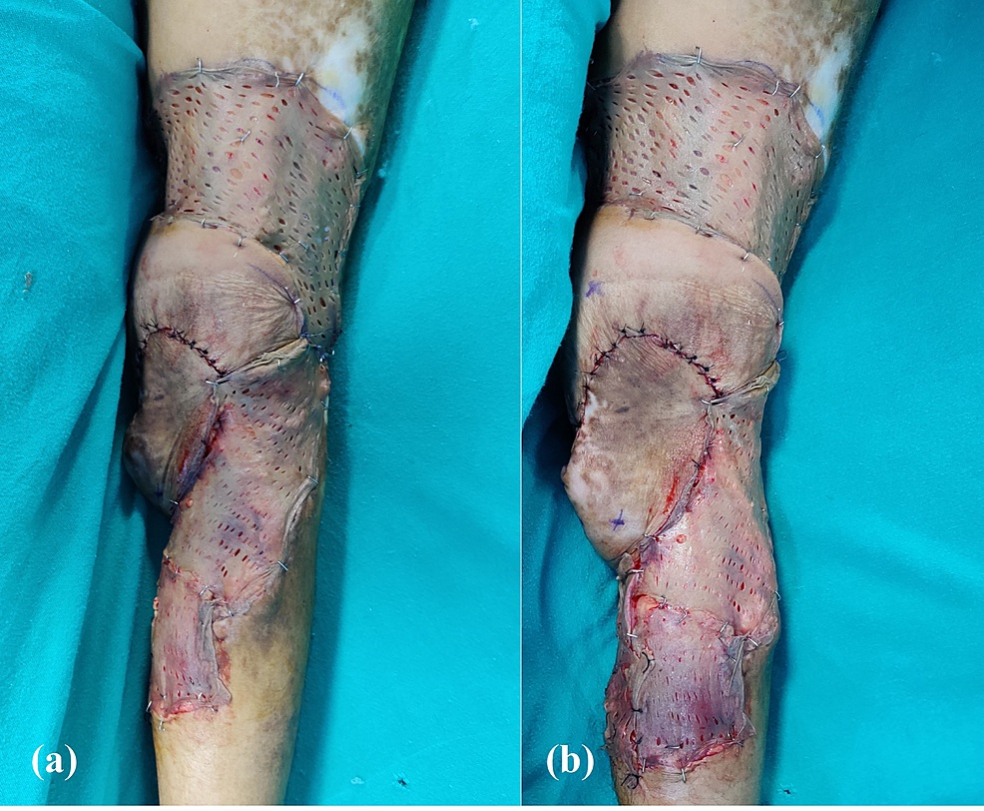
Postoperative photographs of case 1 Clinical photographs showing the successful usage of the boomerang flaps in the defect, post wide local excision (a) both flaps inset over the defect in the right popliteal fossa and skin grafting done for donor sites; (b) markings depicting the perforating vessels over which the flaps were based

A 45-year-old otherwise healthy female presented to us in the outpatient clinic with a history of a postburn scar in the right popliteal fossa. She had suffered a thermal injury 30 years back, which had been managed by dressings and allowed to heal by secondary intention. She had also developed an ulceroproliferative growth in the scar over the past 10 years, which had grown in size and was causing dull pain. On examination, the right popliteal fossa had a large 8 x 6 cm ulceroproliferative swelling with indurated margins. The base of the ulcer was formed by granulation tissue. The scar was extending along the entire posterior aspect of the thigh up to 10 cm below the knee joint. The knee joint could only be extended to 70 degrees due to the contracted skin in the lateral aspect of the popliteal fossa (Figure 1). Intraoperatively, wide local excision of the scar was done in the suprafascial plane, which revealed a defect of 9 x 7 cm in size. No bony or vascular structures were exposed. The knee joint regained full range of movements. Thereafter, local fasciocutaneous flaps were raised based on the inferomedial genicular artery and posterior tibial artery perforators, which had been identified on preoperative Doppler. The two limbs were then rotated into the popliteal fossa and sutured to each other (Figure 2). The secondary defects formed on the lower thigh and upper leg were covered using split-thickness skin grafts harvested from the contralateral thigh.

Case 2

**Figure 3:**
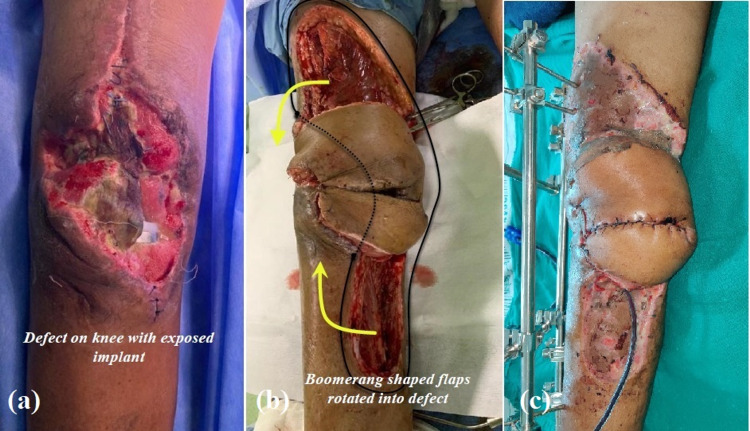
Photographs of case 2 Right knee skin defect (post-surgical site infection) and its coverage with boomerang flaps (a) preoperative defect on the right knee; (b) intraoperative photograph of the flaps after inset; (c) clinical photograph after one week showing healthy flaps and healed donor sites

A 39-year-old male with a history of peripheral arterial occlusive disease in bilateral lower limbs presented to the outpatient clinic with a stiff and painful right knee for one year after having suffered blunt trauma to the right knee. The patient had then developed a swelling over the right knee, which had been treated by incision and drainage. He underwent total knee replacement by the orthopedic team due to septic arthritis. On the fifth postoperative day, the patient developed wound dehiscence due to surgical site infection. The wound was initially managed conservatively with vacuum-assisted closure for seven days. On evaluation after seven days, the wound had improved with the appearance of healthy granulation tissue. However, the implant remained exposed. Post debridement, the patient had a wound present on the anteromedial aspect of the right knee, 14 x 10 cm in size, with the patella and the implant exposed. In view of the non-improving infection, the implant was also removed and the patient was considered for total arthrodesis of the right knee with flap coverage. Fasciocutaneous flaps based on the superior medial genicular artery and medial sural artery were used to resurface the defect. The two limbs of the respective flaps were sutured to each other in a boomerang fashion (Figure 3). The resulting secondary defects were covered using split-thickness skin grafts taken from the left thigh.

Case 3

**Figure 4:**
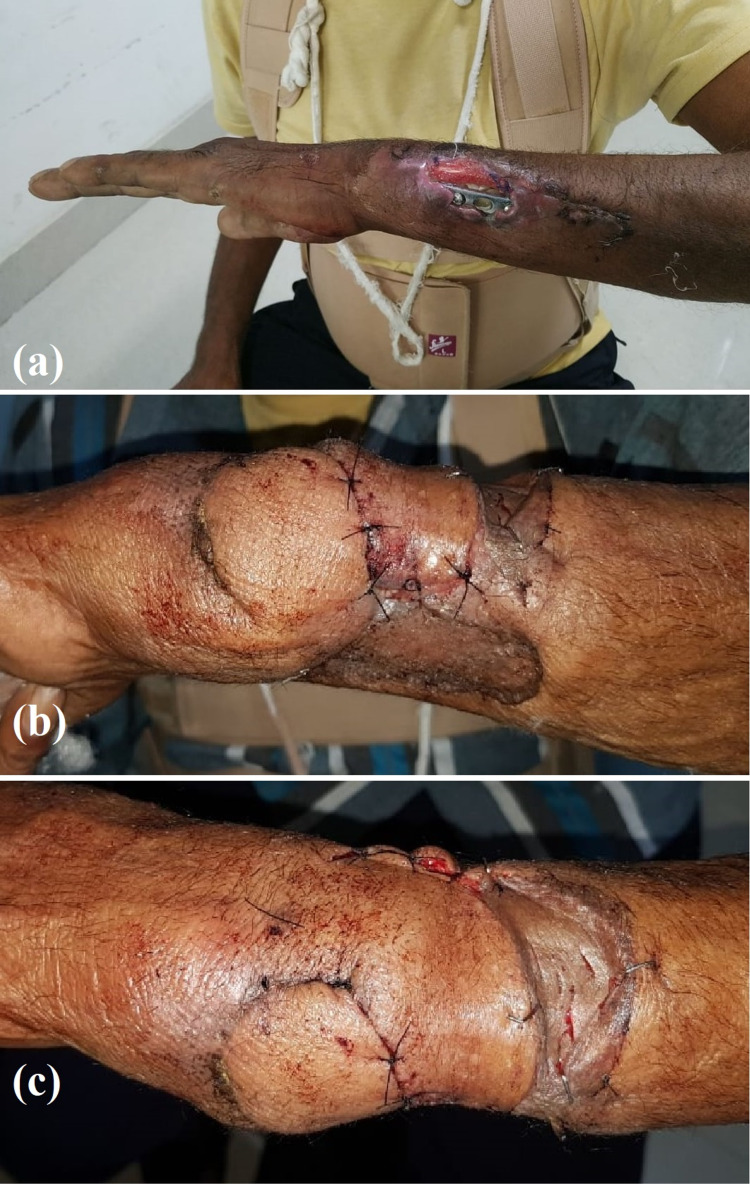
Photographs of case 3 Left forearm soft tissue defect and its coverage using fasciocutaneous flaps (a) preoperative wound on left forearm with implant exposed; (b,c) postoperative clinical photographs showing the two flaps inset in a yin-yang fashion and the donor areas grafted

A 60-year-old otherwise healthy man presented to our department with a history of a fall two months back when he had suffered a fracture of the shaft of the left ulna, which had been managed by open reduction and internal fixation via rigid plating. He had developed wound dehiscence at the operative site and complained of persistent discharge from the wound for the last two months. On examination, there was a 5 x 3 cm wound on the ulnar aspect of the left forearm with the plate exposed. The margins of the wound were sloping and the surrounding skin was scarred. There was no erythema or induration in the local area. Intraoperatively, the wound margins were debrided and two fasciocutaneous flaps were raised based on the septocutaneous perforators of the posterior interosseous branch of the ulnar artery from the anterior and posterior aspects of the ulnar border of the forearm. The flaps were then rotated to cover the defect and sutured with each other in a yin-yang fashion. The resulting secondary defects were covered with split-thickness skin grafts harvested from the thigh (Figure 4).

Case 4

**Figure 5:**
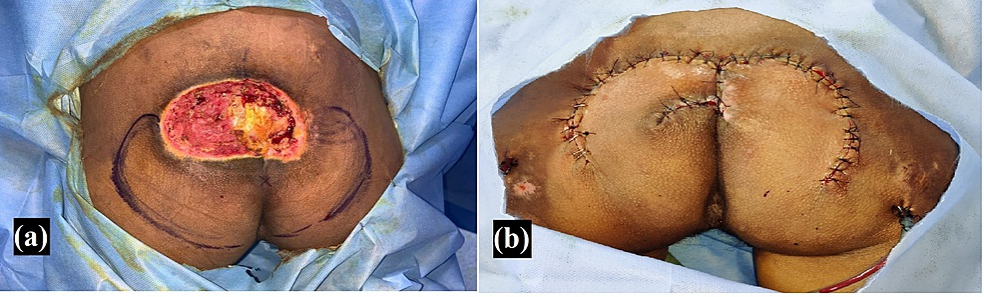
Photographs of case 4 Sacral pressure ulcer and its coverage using fasciocutaneous flaps (a) preoperative image of a grade 4 sacral pressure injury; (b) intraoperative image of the successful usage of the fasciocutaneous flap design utilizing median sacral artery perforator

A 30-year-old female with a history of a fall from height and paraplegia for six months presented to us with a grade IV pressure ulcer over the sacrum. On examination, the ulcer was 10 x 7 cm in dimension with healthy granulation tissue present on the base of the wound. Edges of the ulcer were epithelialized. Intraoperatively, the wound was debrided, and, using a handheld Doppler, a perforator based on the median sacral artery was identified. Keeping the marked perforator at the base, two flaps were raised from either side of the defect and transposed into the defect. The two flaps were then rotated in a boomerang fashion and sutured to each other. By mobilizing the tissue on either side of the secondary defects, we were able to primarily close the secondary defects (Figure 5). This design, apart from being technically simple, also avoided sutures in the zone of pressure and preserved the native tissue vascularity, thereby preventing further chances of pressure ulcers in the patient.

Similarly, the flap design was utilized for four other cases, and the findings are summarized along with those of the above cases in Table 1.

**Table 1:** Summary of our experience

Serial No.	Diagnosis	Defect location	Defect size (cm)	Perforators utilized for the flaps	Secondary defect	Final outcome
1	Post-burn contracture right popliteal fossa with unstable scar	Right popliteal fossa	9 x 7	Inferomedial genicular artery and posterior tibial artery	Split-thickness skin graft	Healthy
2	Post-traumatic right knee defect with exposed implant	Right knee anteromedial aspect	14 x 9	Superomedial genicular artery and medial sural artery	Split-thickness skin graft	Healthy
3	Post-traumatic left forearm defect with exposed implant	Left distal forearm ulnar aspect	5 x 3	Posterior interosseous branch of ulnar artery	Split-thickness skin graft	Healthy
4	Grade 4 pressure ulcer	Sacrum	6 x 6	Median sacral artery	Primary closure	Healthy
5	Left knee post-traumatic defect	Left knee anterior aspect	8 x 4	Inferomedial genicular artery and posterior tibial artery	Split-thickness skin graft	Healthy
6	Post-traumatic right forearm defect	Right distal forearm ulnar aspect	4 x 4	Posterior interosseous branch of ulnar artery	Split-thickness skin graft	Healthy
7	Post-traumatic left forearm defect	Left distal forearm radial aspect	5 x 3	Radial artery	Split-thickness skin graft	Healthy
8	Grade 4 pressure ulcer	Sacrum	7 x 6	Left superior gluteal artery and right superior gluteal artery	Primary closure	Healthy

All cases described here healed with no complications. The donor sites either healed with skin grafting or they were closed primarily. All eight patients were satisfied with the cosmetic outcomes of the procedure.

## Discussion

Fasciocutaneous flaps are the workhorse flaps for most of the soft tissue defects currently. Different modifications like perforator-based [1] and propeller flaps [2] have been described. In a leg with a post-traumatic wound and surrounding scars, the surgeon is forced to use superior and inferiorly based fasciocutaneous flap combinations based on peroneal, posterior tibial, or anterior tibial vessels to reconstruct the defect. This might not be always possible and a free tissue transfer then becomes the only option for such a defect [3]. In this article, we describe a novel design for reconstructing wounds with fasciocutaneous flaps in such circumstances and also explore its use in other regions of the body.

Surgical technique

**Figure 6:**
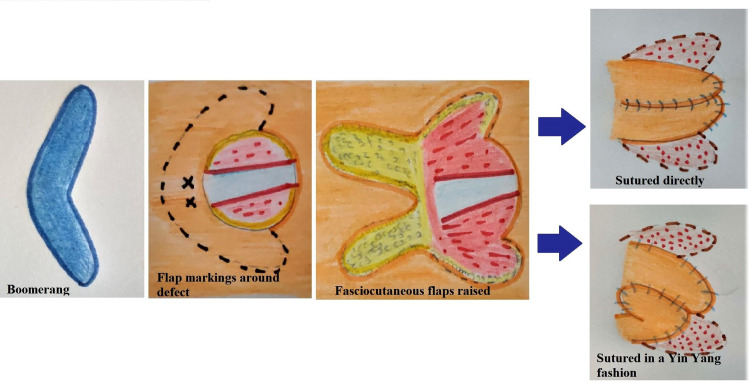
Diagrammatic representation of the surgical technique of our flap (author’s own creation)

This flap is designed in the form of a boomerang, which was used as a traditional hunting tool by Australian tribes. Fasciocutaneous flaps are planned according to the local perforators available in the vicinity of the defect as detected by handheld Doppler preoperatively. The limbs of the flap are raised after ascertaining the perforators in the center of the flap design, with a maximum ratio of 2.5:1 (length: breadth). Then, the raised flaps are advanced into the defect and sutured directly or in a yin-yang manner according to the requirement (Figure 6). In certain cases, if the perforator is well isolated, especially in cases with a single perforator supplying both superior and inferior skin territories, the flap can also be islanded and raised as a propeller flap with limbs capable of independent movement. The resulting secondary defects are then closed primarily or grafted with split-thickness skin grafts according to the pliability of the adjoining tissues.

For leg defects, especially those around the knee joint, the treatments include skin grafting, fasciocutaneous flaps, muscle flaps, and distant flaps with microvascular transfer. Skin grafting is seldom preferred due to its tendency to contract, which can limit joint mobility. The use of a muscle flap like gastrocnemius means losing some function around the knee. Also, the flap being bulky, the patient might not find it aesthetically pleasing [4]. Similarly, other muscle flaps described, such as reversed gracilis flap [5] and sartorius flap [6], are complicated by functional morbidity at the donor site.

Fasciocutaneous flaps, on the other hand, provide a durable and pliable soft tissue cover for the defects including exposed knee joints or implants in that region with minimal donor site morbidity. They also have the advantage of allowing revision surgery at a later time. Various fasciocutaneous flaps have been described to cover defects around knees based on perforators arising from the descending genicular artery [7], perforators of the descending branch of the lateral circumflex femoral artery [8], and lateral superior genicular artery [9]. However, dissection of these flaps can be technically challenging due to the tortuous course of their pedicles. Other fasciocutaneous flaps used include those based on the medial sural artery perforators [10], lateral sural artery perforators [11], lateral genicular artery perforators [12], peroneal artery perforators, posterior tibial, and the anterior tibial artery perforators [13]. However, these flaps may not be always feasible owing to the extent of trauma or previous incisions.

Free flaps like anterolateral thigh flap, latissimus dorsi, and rectus abdominis are viable options in knee reconstruction. However, finding the recipient vessel for microvascular anastomosis poses the greatest challenge apart from the added microsurgical expertise [3].

As we reviewed previously published literature, we found that fasciocutaneous flaps are still the most viable option for knee defects. But when their use is limited by trauma and scarring, there is a requirement for further modification of the existing flap designs according to the donor site availability.

## Conclusions

The authors of this article propose such a modification where the two limbs of a fasciocutaneous flap are raised on the adjacent sides of the defect, based on one or two perforators, in the form of a boomerang design and transposed to the defect. We have used this flap around the knee joint in a case with extensive scarring and have also shown the successful extrapolation of this flap design in other sites including the popliteal fossa region, wrist defect, and sacral pressure ulcer. Due to the technical ease and reliability of the flap, the authors recommend this flap technique when the local tissue availability is not suitable for traditional fasciocutaneous flap designs.
